# Fabrication of 3D-Printed Interpenetrating Hydrogel Scaffolds for Promoting Chondrogenic Differentiation

**DOI:** 10.3390/polym13132146

**Published:** 2021-06-29

**Authors:** Jian Guan, Fu-zhen Yuan, Zi-mu Mao, Hai-lin Zhu, Lin Lin, Harry Huimin Chen, Jia-kuo Yu

**Affiliations:** 1 Beijing Key Laboratory of Sports Injuries, Sports Medicine Department, Peking University Third Hospital, Beijing 100191, China; guanjian@bjmu.edu.cn (J.G.); 1916384020@bjmu.edu.cn (F.-z.Y.); maozimu@yeah.net (Z.-m.M.); Simon.lin2003@163.com (L.L.); 2 Institute of Sports Medicine of Peking University, Beijing 100191, China; 3 SinoBioPrint (Shanghai) Biotech Ltd., 23 Building, 1188 Lianhang Road, Minhang District, Shanghai 201112, China; hailin.zhu@stemeasy.com

**Keywords:** 3D printing, hydrogel scaffold, cartilage repair, GelMA, CSMA

## Abstract

The limited self-healing ability of cartilage necessitates the application of alternative tissue engineering strategies for repairing the damaged tissue and restoring its normal function. Compared to conventional tissue engineering strategies, three-dimensional (3D) printing offers a greater potential for developing tissue-engineered scaffolds. Herein, we prepared a novel photocrosslinked printable cartilage ink comprising of polyethylene glycol diacrylate (PEGDA), gelatin methacryloyl (GelMA), and chondroitin sulfate methacrylate (CSMA). The PEGDA-GelMA-CSMA scaffolds possessed favorable compressive elastic modulus and degradation rate. In vitro experiments showed good adhesion, proliferation, and F-actin and chondrogenic differentiation of bone marrow mesenchymal stem cells (BMSCs) on the scaffolds. When the CSMA concentration was increased, the compressive elastic modulus, GAG production, and expression of F-actin and cartilage-specific genes (COL2, ACAN, SOX9, PRG4) were significantly improved while the osteogenic marker genes of COL1 and ALP were decreased. The findings of the study indicate that the 3D-printed PEGDA-GelMA-CSMA scaffolds possessed not only adequate mechanical strength but also maintained a suitable 3D microenvironment for differentiation, proliferation, and extracellular matrix production of BMSCs, which suggested this customizable 3D-printed PEGDA-GelMA-CSMA scaffold may have great potential for cartilage repair and regeneration in vivo.

## 1. Introduction

The degeneration of articular cartilage due to trauma, osteoarthritis, and aging is a common joint disorder worldwide [1]. It is reported that the limited intrinsic self-healing capacity was noted on cartilage because of its avascular nature [2]. Although some clinical treatments, such as autologous chondrocyte implantation mosaicplasty and microfracture, are available for articular cartilage repair [3], such techniques are limited in offering long-term correction of cartilage pathologies [4], thus necessitating the development of alternative strategies. The development of synthetic scaffolds via tissue engineering approaches can be a promising strategy for cartilage regeneration and repair [5,6].

The application of hydrogels is receiving increasing consideration in biomedical fields, including drug delivery, tissue engineering, and regenerative medicine. Additionally, the hydrogels possess similar morphology to the natural extracellular matrix (ECM), offering mimetic conditions for cell culture [7,8]. Three-dimensional (3D) printed scaffolds have already been proven as better choices for cartilage tissue engineering than those conventional techniques such as freeze-drying [9]. 3D printing using computer-aided design (CAD) has the potential to precisely regulate the uniform morphology and interconnected porous structure of bioinspired scaffolds, which provides the base for fabricating complex, customizable, and artificial constructs for cartilage regeneration [10]. Fused deposition modeling (FDM) is an additive manufacturing technique used for prototyping, production, and modeling applications. FDM enables the rapid fabrication of highly interconnected pore geometries and channel sizes [11,12]. Moreover, it is a cost-effective approach to print high-resolution constructs compared to other 3D printing techniques, which are often overwhelmed by the viscosity of bio-ink.

Gelatin methacrylate (GelMA) is a major component of ECM derived from the hydrolysis of collagen, which contains matrix metalloproteinase sequences and arginine-glycine-aspartic (RGD) acids for promoting cell adhesion and proliferation [13]. Chondroitin sulfate (CS), a natural polymer, is predominantly part of the aggrecan. A previous study suggested that CS mediated several biological mechanisms involved in developing resistance to compressive loading [14]. It was found that CS-mediated expression of chondrocytes phenotype was useful for guiding the tissue repair for regenerative medicine applications [15]. Meanwhile, another photocrosslinkable biomaterial of poly (ethylene glycol) diacrylate (PEGDA) was blended with GelMA and chondroitin sulfate-methacrylamide (CSMA) for improving the printability and strength of the scaffolds [16]. The aforementioned three kinds of photocurable biomaterials can be well-polymerized under 405 nm blue light [17,18,19].

In the present study, a novel ink comprising of PEGDA, GelMA, and CSMA was prepared to print 3D scaffolds for cartilage tissue regeneration. We used the conventional FDM to print a poly (lactic acid) (PLA) porous scaffold as a mold, followed by mixing gel precursors and pouring it into the mold, leaving the liquid level a little above those. After exposure to 405 nm blue light for 30 s and removal of pre-mold, high-resolution 3D-printed PEGDA-GelMA-CSMA hydrogel scaffolds were obtained with uniform interconnected pores. The 3D-printed interpenetrating polymer network (IPN) hydrogel scaffold was fabricated for promoting the chondrogenic differentiation of BMSCs. BMSCs were seeded on different 3D-printed PEGDA-GelMA-CSMA scaffolds, and the relevant physicochemical and biological characteristics of the 3D scaffolds were investigated. Additionally, the expression of cartilage-specific marker genes, main osteogenic marker genes, and secretion of glycosaminoglycan (GAG) were also determined for evaluating the chondrogenic differentiation of BMSCs on the 3D scaffolds in vitro. These PEGDA-GelMA-CSMA hydrogel scaffolds mimicked ECM composition and demonstrated the biomechanical properties of cartilage tissue by providing an appropriate microenvironment for exogenous seeded cells or endogenic BMSCs. We believe this study will provide a platform for designing the 3D-printed hydrogel scaffolds as a promising one-step approach by combining the endogenic and exogenous BMSCs for cartilage repair.

## 2. Materials and Methods

### 2.1. Materials

Gelatin methacrylate (GelMA), chondroitin sulfate-methacrylamide (CSMA) and the photoinitiator, lithium phenyl-2,4,6-trimenthylbenzoylphosphinate (LAP) were obtained from SinoBioPrint (Shanghai, China) Biotech Ltd (Shanghai, China).

### 2.2. Designing of Mold

A 3D CAD model of 45 × 45 mm^2^ grid-network structure (3 mm in height) was designed via CREO (STL file) and Repetier-Host (print parameters) soft wares (Hot-World GmbH & Co. KG, Willich, Germany). We chose a common thermoplastic filament PLA for FDM printing [12]. The 3D printing system used in this study was a commercial desktop printer (JGAURORA, 305 mm × 305 mm × 320 mm, Shenzhen, China). The printing parameters were set as speed 60 mm/s, nozzle temperature 210 °C, platform temperature 60 °C, FDM printer nozzle diameter 250 μm, and grid model infill ratio 50%.

### 2.3. Synthesis of Photo-Polymerizable Mixture Gel Precursor

GelMA and CSMA were synthesized according to previous studies [20,21]. PEGDA (M_n_ = 700, Sigma-Aldrich, St. Louis, MA, USA) was mixed with different amounts of GelMA and CSMA. The photoinitiator of lithium phenyl-2,4,6-trimethylbenzoylphosphinate (LAP) was used to form the photo-polymerizable mixture gel precursor by dissolving in deionized water at 60 °C.

### 2.4. Preparation of Photocurable IPN Hydrogel Porous Scaffolds Using 3D Composite-Printing and Lyophilization

The aforementioned gel precursors were poured into the mold, leaving the liquid level a little above that, and then the composites were exposed to a wavelength of 405 nm blue light laser source (30 mW/cm^2^) for 30 s to form photocurable hydrogel (irreversible). The composites were soaked in the dichloromethane solution for several hours, during the process, the PLA mold would be dissolute gradually, then remove the waste liquid, and soaked in deionized water for a few minutes. Finally, the remaining reinforced photocurable IPN hydrogel porous scaffolds were cut into different sizes by specific corneal trephine. After preparation, the scaffolds were frozen at −20 °C for 2 h and then lyophilized for 12 h. The scaffolds were sterilized with ethylene oxide and the experiment was started.

### 2.5. Characterization of Macromers and 3D-Printed Hydrogel Scaffolds

Gelatin, GelMA, CS, and CSMA were characterized and analyzed by ^1^H NMR (Bruker 400MHz NMR spectrometer). The morphology of the 3D printing scaffold was observed through an optical microscope and scanning electron microscope (SEM). The samples were plated about 8 nm in a high vacuum gold sputter coater and observed under a Phenom Pharos SEM (Holland). The universal testing machine with 6 mm/min as a crosshead speed was applied to test the mechanical properties of 3D scaffolds. Briefly, the samples were cut into 10 mm diameter and 3 mm thick disks. The stress-strain curve was used for calculating the compressive elastic modulus. All analyses were performed in triplicate.

### 2.6. In Vitro Degradation

The degradation behavior of 3D-printed PEGDA-GelMA-CSMA hydrogel scaffolds was evaluated by recording the weight loss of scaffolds in proteinase K solution over 21 days. We recorded the initial weight of lyophilized PEGDA-GelMA-CSMA hydrogel scaffolds (*W*_0_). The scaffolds were incubated in proteinase K for 1, 4, 7, 14, and 21 days at 37 °C. After incubation, the scaffolds were lyophilized and weighed again (*W*_t_). The degradation ratio was calculated as the following:

Degradation ratio (%) = (*W*_0_ − *W*_t_)/*W*_0_ × 100%

### 2.7. Isolation and Culturing of BMSCs

The Animal Care and Use Committee of Peking University Third Hospital approved all the protocols of animal experiments that were implemented following the Guide for the Care and Use of Laboratory Animals. BMSCs were isolated following the protocol reported in a previous study [3]. At a confluence of 80−90%, BMSCs were trypsinized with 0.25% trypsin/0.1% ethylene diamine tetra-acetic acid for subculture at 1:2. BMSCs were cultured in a-MEM with penicillin (100 U/mL), streptomycin (100 U/mL), and 15% fetal bovine serum. In the chondrogenic differentiation study, the BMCs-seeded constructs were cultured in a chondrogenic differentiation medium (CTCC-Y002; CTCC Biosciences Inc., Jiangyin, China) after three days in the growth medium.

### 2.8. Cell Seeding on 3D Hydrogel Scaffolds

A 10 μL of BMSCs suspension at a density of 1 × 10^7^ cells/mL was seeded on 3D-printed hydrogel scaffolds of 5 mm diameter and 3 mm thickness by centrifugation [22]. The cell-scaffold composites were incubated for 1 h to allow the adhesion of cells, and then the cell-seeded hydrogel scaffolds were transferred to 96-well plates for suspension culture, followed by the addition of 150 μL of fresh growth medium.

### 2.9. Cytocompatibility

Cell proliferation on scaffolds was detected by cell counting kit-8 (CCK-8; Dojindo Laboratories, Kumamoto, Japan). The original culture medium was replaced with 90 µL of fresh culture medium and 10 µL CCK-8 reagent after incubation for 1, 3, 5, and 7 days. The absorbance was detected at 450 nm every 2 h using a microplate reader (Thermo, Waltham, MA, USA).

### 2.10. Live/Dead Staining

To visualize cell growth and distribution on hydrogel scaffolds, a Live/Dead staining assay was performed by the Viability/Cytotoxicity Kit (Invitrogen, Carlsbad, CA, USA). After incubation for 3 days, the cell-hydrogel scaffold composites were washed and immersed in 2 mM of calcein-AM and 4 mM ethidium homodimer-1 for 2 h at 37 °C. The fluorescence was observed by using Leica TCS-SP8 confocal laser microscopy (CFLM; Leica, Nussloch, Germany) at an excitation wavelength of 568 or 488 nm. Imaris software 7.4.2 (Bitplane, Oxford, UK) was applied to observe the distribution of BMSCs on the hydrogel scaffolds.

### 2.11. Cytoskeleton Staining

Cytoskeleton staining was performed to observe the morphology of cells on 3D hydrogel scaffolds. After incubation for 5 days, the scaffolds were fixed in 4% paraformaldehyde for 30 min after washing. Thereafter, the cells on scaffolds were treated with 0.1% Triton X-100 solution in PBS and then subjected to F-actin staining and DAPI staining for 1 h and 5 min, respectively. Finally, the fluorescence microscope (Leica, TCS SP-8, Wetzlar, Germany) was used to observe the samples.

### 2.12. RT-PCR Test

TRIZOL reagent was used to extract the total RNA (Invitrogen, Carlsbad, CA, USA) when the cell-hydrogel composites were incubated for 7 and 14 days. The RNA was reverse transcribed into cDNA by MMLV Reverse Kit, and RT-PCR was conducted by Real-time PCR system (Applied Biosystems, Waltham, MA, USA) with SYBR Green PCR Master Mix (Toyobo, Japan). The value of relative expression of genes was plotted as 2^−ΔΔCT^ as reported previously [3]. The primers used for the amplification of target genes were listed in Table 1.

### 2.13. Immunofluorescence Staining

The production of COL2, SOX9, and ACAN proteins in cell-scaffold constructs was determined by immunofluorescence after three weeks of chondrogenic culturing. The constructs were washed with PBS after fixation with 4% paraformaldehyde for 30 min. Thereafter, the constructs were incubated with 10% FBS for 60 min at 37 °C followed by incubation with a solution of primary antibody for anti-collagen type 2 (Abcam, Cambridge, UK, ab34712), SOX9 (Abcam, ab185966), and ACAN (Proteintech, Chicago, IL, USA, 13880-1-AP) for 24 h at 4 °C. The nuclei were counter-stained with Hoechst 33258. The proportion of cells expressing the COL2, ACAN, and SOX9 was analyzed by the Image-Pro Plus software (6.0; Media Cybernetics, Rockville, MD, USA).

### 2.14. Quantification of DNA, GAG, and COL2 Content

The COL2 content was tested by an ELISA kit following the manufacturer’s instructions (Jianglai bio, Shanghai, China, JL22853). The content of proteoglycan was detected from GAG content by the 1, 9-dimethyl methylene blue (DMMB; Sigma, St. Louis, MO, USA) dye-binding assay. Total GAG was normalized to total DNA content. Thereafter, 20 μL of the sample was added to DMMB (200 μL) and mixed, and the absorbance was measured at 525 nm. A standard curve was established from chondroitin-6-sulfate, using shark (Sigma, St. Louis, MO, USA). A fluorometric assay was performed to detect DNA content. Specimens were digested in a mixed solution of EDTA (0.5 M), Mcysteine-HCl (0.05), and papain enzyme (1 mg/mL) at 70 °C for 48 h after weighing. Aliquots of sample digestion were stained with 200 μL of Hoechst33258 (2 μg/mL) at 37 °C for 20 min. The fluorescence intensities were then determined at an excitation wavelength (360 nm) and an emission wavelength (460 nm). The DNA content was normalized with a standard curve of calf thymus DNA (Sigma, St. Louis, MI, USA).

### 2.15. Statistical Analysis

The statistical data were expressed as mean ± standard deviation (SD). One-way analysis of variance (ANOVA) was performed for determining the differences among the groups after testing for homogeneity of variances. *p* < 0.05 was considered statistically significant. GraphPad Prism for Windows (GraphPad Software, San Diego, CA, USA) was used to analyze the data.

## 3. Results

### 3.1. Preparation and Characterization of GelMA and CSMA

**Figure 1:**
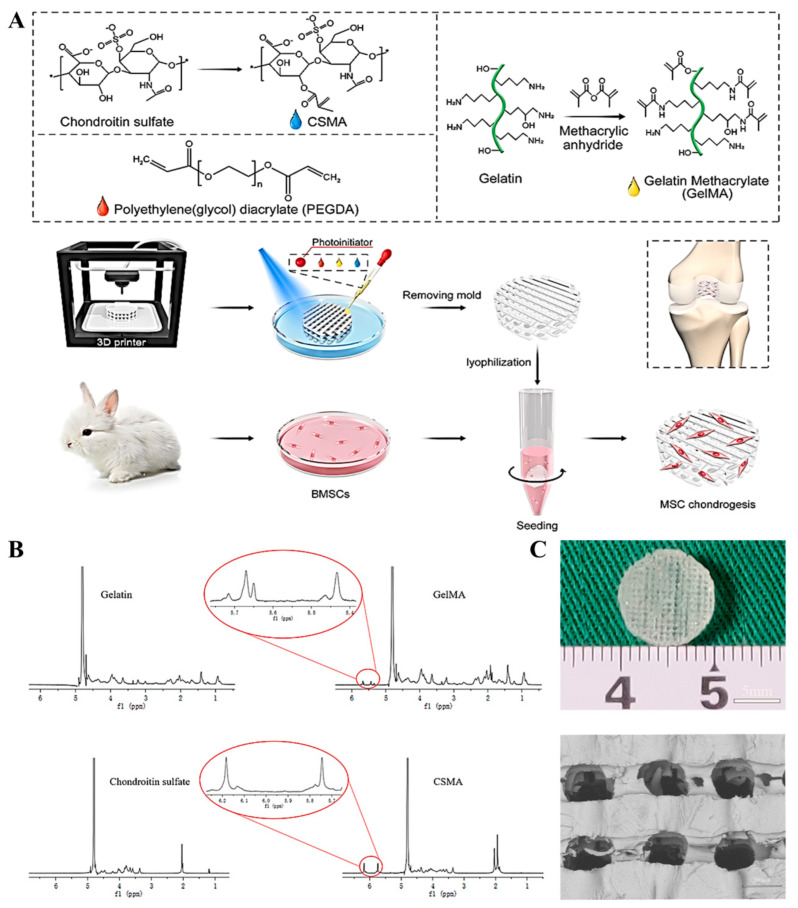
Fabrication of 3D-printed hydrogel scaffolds. (**A**) Synthetic route of CSMA and GelMA and the schematic illustration of 3D printing of scaffolds as well as the isolation and seeding of cells on the printed scaffolds. (**B**) ^1^H-NMR spectra of GelMA and CSMA. (**C**) Macroscopic and SEM images of the 3D spatial structure of scaffold.

Figure 1A showed the schematic illustration of preparation and biological evaluation of 3D-printed scaffolds for cartilage tissue regeneration application. ^1^H NMR spectra analysis was used to testify the synthesis of GelMA and CSMA. The methacrylamide vinyl group signal occurred at 5.5 and 5.7 ppm after adding MA into gelatin (Figure 1B), suggesting the successful grafting of the methyl acryloyl group onto the side chain of gelatin. Similarly, the methacrylamide vinyl group signal at 5.7 and 6.2 ppm indicated the successful preparation of CSMA (Figure 1B). The 3D-printed PEGDA-GelMA-CSMA hydrogel scaffolds with uniformly interconnected pores and high resolution were fabricated with 3D printing by removing the pre-mold. The hydrogel scaffolds were fabricated via FDM with dimensions of 45 × 45 × 3 mm^3^ by removing the mold and trimmed into cylindrical samples with a 5-mm diameter corneal trephine. The resultant scaffolds of 3 mm thickness and 5 mm diameter were obtained in Figure 1A. A pore size between 100 to 400 μm in cartilage repair is better for proliferation, differentiation, and ECM production. Therefore, these 3D-printed scaffolds possessed a smooth surface, uniform pores (200 μm), and well-arranged channels (Figure 1C). These observations showed that the 3D-printed scaffolds with the hierarchical structure were successfully prepared via the 3D composite-printing strategy.

### 3.2. Mechanical Properties and Biodegradation of 3D-Printed Hydrogel Scaffolds

**Figure 2:**
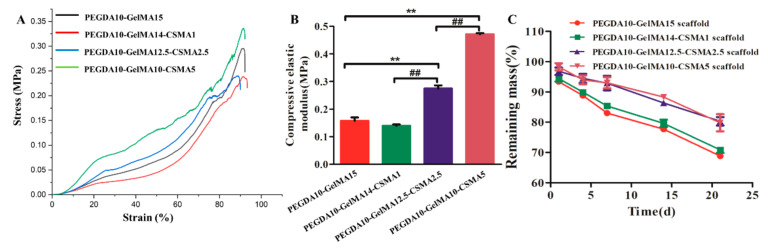
The mechanical properties and biodegradation of 3D-printed hydrogel scaffolds. (**A**) Stress-strain curves. (**B**) Compressive elastic modulus (*n* = 3, ** *p* < 0.01, ^##^ *p* < 0.01). (**C**) In vitro degradation of scaffolds in protease K at each time point (*n* = 5).

In the present study, four types of scaffolds—made by mixing PEGDA solution (*w*/*v*), GelMA (*w*/*v*), and CSMA (*w*/*v*)—were designed at various mass ratios of 10/15/0, 10/14/1, 10/12.5/2.5, and 10/10/5, respectively (Table 2). A compression measurement was performed to assess the compressive stiffness of 3D-printed hydrogel scaffolds in Figure 2A. The elastic modulus was detected by the applied force normalized to the sample cross-sectional area divided by the compressive strain. The results showed that the trend of stress and compressive modulus was consistent (Figure 2A,B). Compared to the PEGDA-GelMA scaffold, the level of stress and compressive modulus was significantly higher in the PEGDA10-GelMA12.5-CSMA2.5 and PEGDA10-GelMA10-CSMA5 scaffolds (*n* = 3, *p* < 0.01).

The biodegradation of scaffolds was tested in protease K at 37 °C (Figure 2C). The incorporation of CSMA into the scaffolds increased the cross-linked density and internal structural compactness. Therefore, the scaffolds containing CSMA exhibited a slower degradation rate than the hydrogels without CSMA. After the incubation of 21 days, the remaining masses were 68.82 ± 0.67% for PEGDA10-GelMA15 scaffold, 70.84 ± 0.99% for PEGDA10-GelMA14-CSMA1 scaffold, 80.30 ± 1.40% for PEGDA10-GelMA12.5-CSMA2.5 scaffold, and 79.80 ± 2.83% for PEGDA10-GelMA10-CSMA5 scaffold. The incorporation of CSMA was useful to maintain the structural stability of hydrogel scaffolds. Structurally stable hydrogels would be beneficial to the proliferation, adhesion, and ECM production of cells.

### 3.3. Cytocompatibility of 3D-Printed Scaffolds

**Figure 3:**
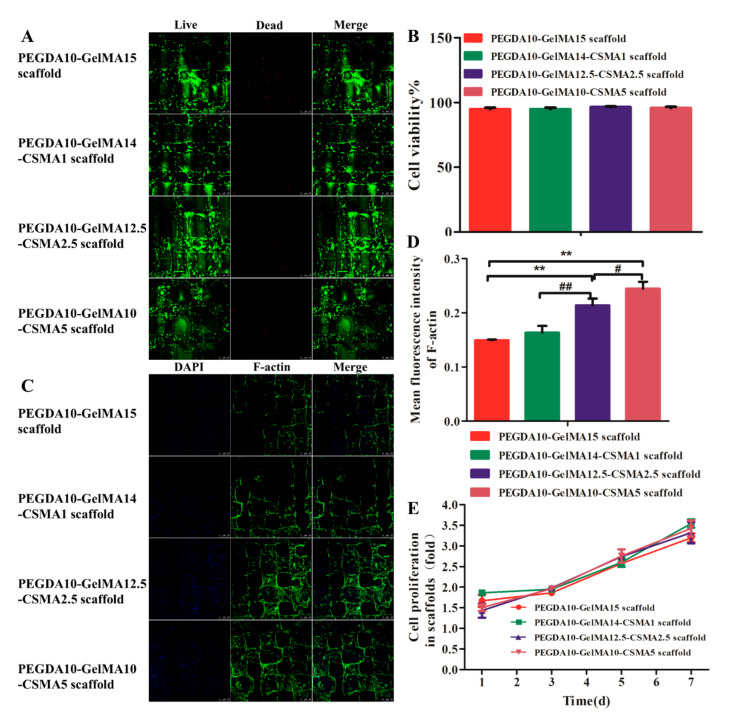
The cytocompatibility of 3D-printed scaffolds. (**A**) Live/dead staining images. (**B**) Percentage of live cells detected by Live/Dead staining. The live cells are indicated by green staining, while dead cells are shown as red. (**C**) Cytoskeletal staining images. (**D**) mean fluorescence intensity of F-actin. (**E**) CCK-8 assay. The scale bar is 25 µm (*n* = 3; ** *p* < 0.01, ^##^ *p* < 0.01, ^#^ *p* < 0.05).

The Live/Dead assay suggested that most of the seeded cells (95%) survived on the scaffolds, with only a few dead cells found after 72 h of culture in the growth medium (Figure 3A). These results showed the low cytotoxicity of four biocompatible hydrogel scaffolds. After seven days of culture, the viability of BMSCs in the PEGDA-GelMA scaffold was similar to that of PEGDA-GelMA-CSMA scaffolds (*n* = 3, *p* > 0.05) (Figure 3B). The cytoskeleton staining was carried out to show the morphology of BMSCs in tested scaffolds after culturing for 72 h as shown in Figure 3C. The mean fluorescence intensity indicated that the expression of F-actin of BMSCs was significantly upregulated with the increasing content of CSMA (*n* = 3, *p* < 0.05) (Figure 3D). Therefore, the CCK-8 assay indicated the proliferation of BMSCs on the four groups of scaffolds had no statistical difference among them at day 1, 3, 5, and 7 (*n* = 5, *p* > 0.05) (Figure 3E).

### 3.4. RT-PCR Analysis

**Figure 4:**
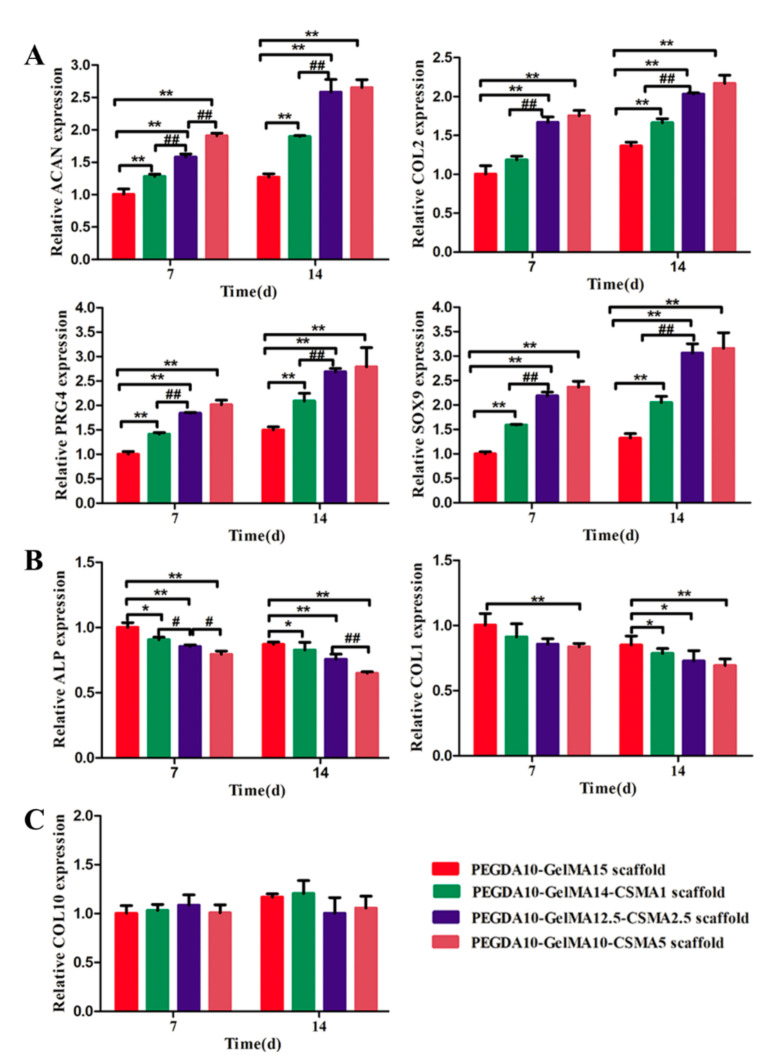
RT-PCR analysis. (**A**) The expression of cartilage-specific genes (ACAN, COL2, SOX9, and PRG4) and (**B**) osteogenesis marker gene (ALP and COL-1). (**C**) hypertrophic gene COL-10 of BMSCs within various scaffolds determined by RT-PCR tests on day 7 and 14 (*n* = 3; * *p* < 0.05, ^#^ *p* < 0.05, ** *p* < 0.01, ^##^ *p* < 0.01).

The effect of CSMA on chondrogenic differentiation of BMSCs on the scaffolds was investigated by evaluating the expression of cartilage-specific genes, including COL-2, SOX-9, ACAN, and PRG4 at mRNA level as shown in Figure 4A. We analyzed the expression of osteogenesis marker gene ALP and COL-1 and hypertrophic gene COL-10 (Figure 4B,C). Compared to the PEGDA-GelMA scaffold, the expression of COL-2, SOX-9, ACAN, and PRG4 were significantly upregulated, while the expression of ALP and COL-1 were significantly downregulated in the PEGDA-GelMA-CSMA scaffolds after incubation for 7 and 14 days (*n* = 3, *p* < 0.05). There was no significant difference in the expression of COL-10 in the tested groups (*n* = 3, *p* > 0.05), suggesting that four scaffolds could support cell growth and prevent cell aging. These results showed that the expression of COL-2, SOX-9, ACAN, and PRG4 and osteogenesis marker genes (ALP and COL-1) changed significantly with the increasing concentration of CSMA in the indicated scaffolds.

### 3.5. COL 2, ACAN, and SOX 9 Production Analysis by Immunofluorescence

**Figure 5:**
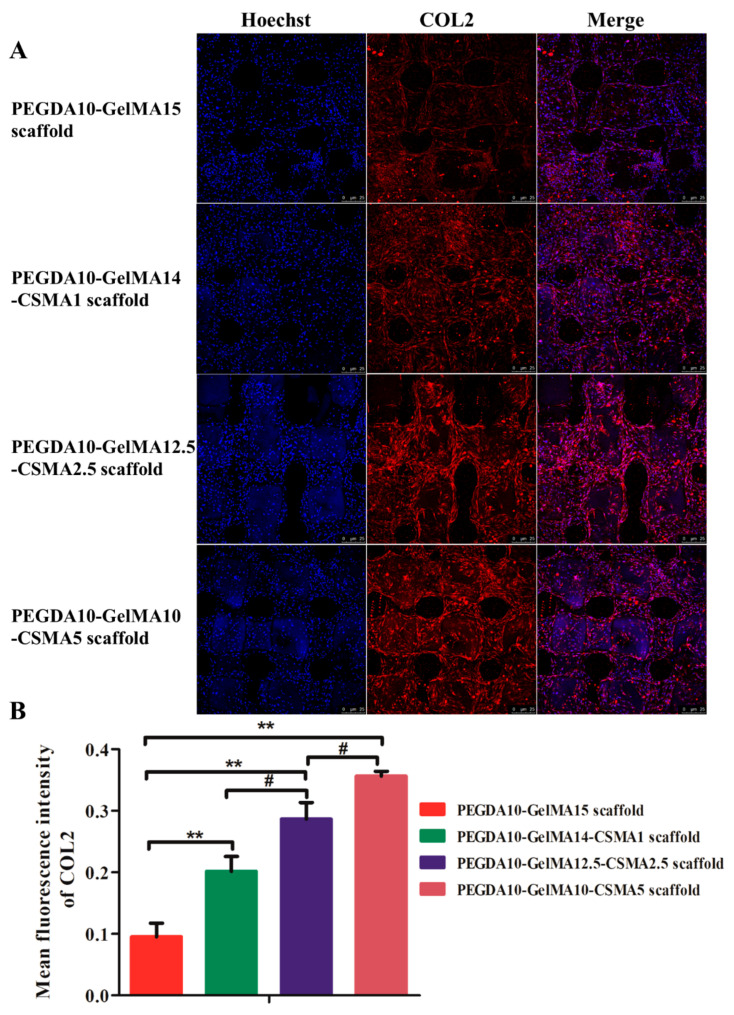
Immunofluorescent staining of COL2 on the scaffolds seeded with BMSCs. (**A**) Immunofluorescence staining images and (**B**) mean fluorescence intensity of COL2 secreted by BMSCs on four scaffolds on day 14. The nuclei are stained blue while the protein COL2 is stained red. The scale bar is 25 µm (*n* = 3; ** *p* < 0.01, ^#^ *p* < 0.05).

**Figure 6:**
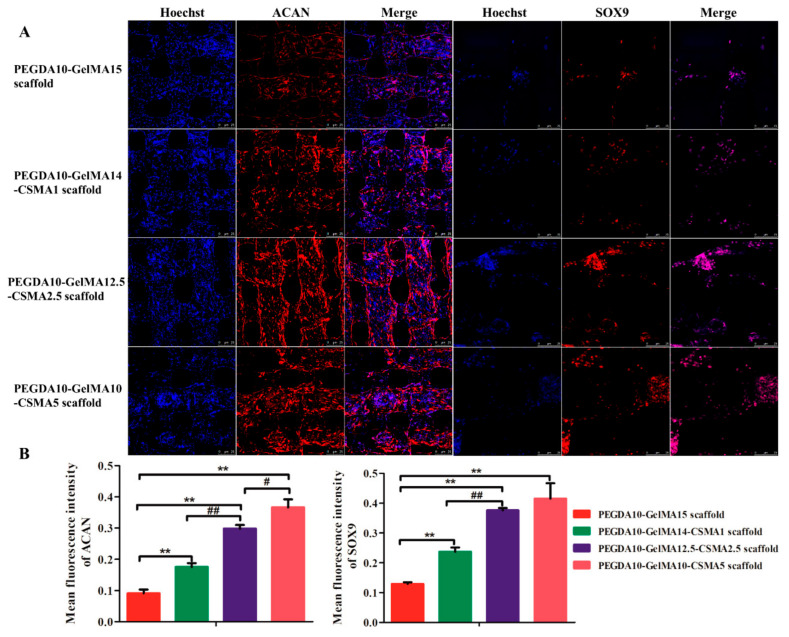
ACAN and SOX9 production analysis by immunofluorescence. (**A**) Immunofluorescence staining images and (**B**) fluorescence intensity of ACAN and SOX9 secreted by BMSCs on four scaffolds on day 14. The nuclei are stained blue. The proteins ACAN and SOX9 are stained red. The scale bar is 25 µm (*n* = 3; ** *p* < 0.01, ^##^ *p* < 0.01, ^#^ *p* < 0.05).

BMSCs are an essential and multipotent form of stem cell which can undergo osteogenic, chondrogenic, and adipogenic differentiation under specific conditions. In the present study, the chondrogenic differentiation was induced on four types of scaffolds for two weeks. The expression profiles of the cartilage-specific markers (COL2, ACAN, and SOX9) were evaluated by immunofluorescence staining. Figure 5 and Figure 6 showed that after 14 days of culture, the expression of COL2, ACAN, and SOX9 was significantly increased in the PEGDA-GelMA-CSMA scaffolds compared to the PEGDA-GelMA scaffold. These results indicate that the addition of CSMA to the scaffolds could remarkably enhance the chondrogenic differentiation of BMSCs.

### 3.6. ECM Deposition on Scaffolds

**Figure 7:**
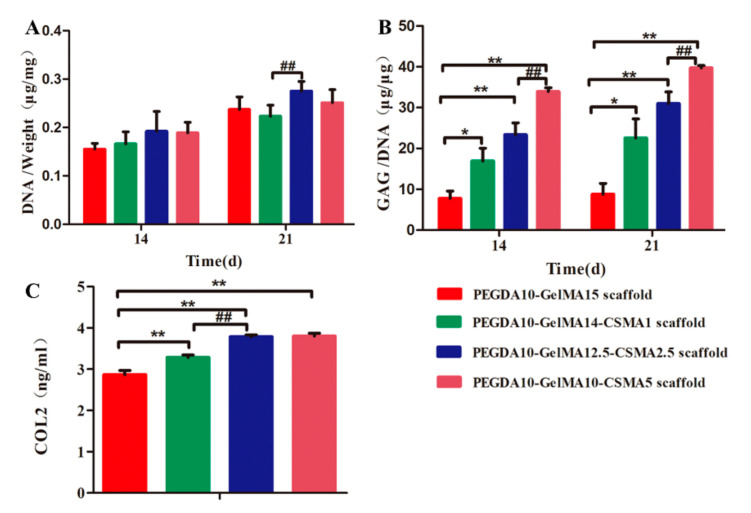
DNA contents, GAG production, and COL2 proteins in different types of PEGDA-GelMA-CSMA scaffolds on day 14 and day 21 culture. (**A**) DNA contents, (**B**) GAG production, and (**C**) COL2 deposition on four scaffolds by the BMSCs (*n* = 3; * *p* < 0.05, ** *p* < 0.01, ^##^ *p* < 0.01).

The DNA contents of four cell-hydrogel composites increased over time. The average DNA content in groups of PEGDA-GelMA-CSMA scaffolds was higher than the PEGDA-GelMA scaffold, but no statistical difference was noted among the tested groups (*n* = 3, *p* > 0.05) (Figure 7A). On days 14 and 21, a significantly higher GAG content was found in the PEGDA-GelMA-CSMA scaffold groups than in the PEGDA-GelMA group (Figure 7B). In addition, COL-2 content in four hydrogel scaffolds was quantitatively detected for 3 weeks culture by ELISA kit (Figure 7C). The content of COL2 in tested scaffold groups was significantly increased after incubation of 21 days, which was consistent with the GAG production trend detected by the DMMB (*p* < 0.01). The fraction of total GAGs was influenced by the presence of CSMA. GAG was an essential ECM component in articular cartilage, and its production was directly correlated with the level of chondrogenesis. Therefore, these results suggested that the addition of CSMA to the scaffold could remarkably enhance the chondrogenic differentiation of BMSCs.

## 4. Discussion

Tissue engineering is considered for obtaining tissue reconstruction through biomaterials and the external seeding of cells. However, considering the time and cost of harvesting, proliferation, and differentiation of cells, a cell-free scaffold-based strategy could be of high clinical value for tissue regeneration and repair [23,24,25,26]. Currently, 3D printing technologies are receiving immense consideration for the fabrication of tissue scaffolds with well-defined microarchitecture for specific applications. Some 3D printing technologies allow cell-loaded printing, which can facilitate tissue regeneration by significantly enhancing the interaction between the cells and scaffold matrix. Similarly, the hydrogels as the substrate with direct contact with the cells (in vitro) and tissues (in vivo) should be biocompatible, non-toxic, and biodegradable in the formulation of bio-ink. To date, 3D bioprinting of scaffolds has been achieved for different applications, especially for cartilage regeneration [27,28]. The hydrogel-based bio-inks could be used as a suitable scaffold material for cartilage regeneration because of their structural similarity to ECM, high water retention, and biocompatibility [29,30]. Considering the abundance of CS as GAGs in cartilage ECM, it was added into the hydrogels to obtain the scaffolds with a biomimetic environment for enhancing chondrogenesis [31]. In addition, GelMA was also a biocompatible agent with photocrosslinking ability to be widely applied in cell-laden bioprinting [32]. To improve the hydrogel scaffolds with high mechanical strength, CSMA and GelMA with a high content of double bond were synthesized for the structural crosslinking (Figure 1). 3D printing can accurately determine the external and internal pore structure of the scaffold. In this study, the 3D printing technique was employed for the fabrication of a hydrogel cartilage construct by mixing PEGDA, GelMA, and CSMA. Accurate control of internal pore size is another key requirement of porous scaffold [33]. In macroporous scaffolds, porosity can guide tissue regeneration by regulating vascularization [34]. The reports suggest that the optimal pore size of the scaffolds ranges from 100 to 400 µm [35,36,37]. In the present study, PEGDA-GelMA and PEGDA-GelMA-CSMA scaffolds provided a uniform pore size of 200 µm (Figure 1C). Besides, sufficient interconnectivity of porous hydrogel scaffolds is also important for nutrient uptake, exchange of gases, cell migration, and cell-cell interaction, which is crucial for chondrogenesis.

The scaffold with suitable mechanical properties mimicking the native tissues is one critical 3D scaffold design criterion. Although CSMA and GelMA are highly biocompatible materials for cell-laden bioprinting, their mechanical properties and printing resolution greatly limited their application in 3D bioprinting [32]. In the present study, we added CSMA into the PEGDA-GelMA hydrogels, which greatly enhanced the compressive elastic moduli in PEGDA10-GelMA12.5-CSMA2.5 and PEGDA10-GelMA10-CSMA5 scaffolds compared to the pure PEGDA-GelMA (Figure 2A).

Some GelMA hydrogels were reported to degrade in a few hours in vitro [38,39,40], while others remain stable even up to 10 days [41]. The degradation experiments lasted for several weeks when no enzyme was added to the system [42,43]. It was reported that cell-secreted ECM and the hydrogels were sensitive to enzymatic degradation reaction [44,45]. Thus, the mechanical properties of the constructs were associated with the balance between anabolic and catabolic factors combined with hydrolytic degradation. The biodegradation tests performed in the solution of proteinase K indicated that there was no significant change in the biodegradation rate among the tested scaffolds (Figure 2). Therefore, the results of biodegradation were consistent with the uniform porous structure of the 3D-printed PEGDA-GelMA-CSMA porous scaffold. The biodegradation was closely related to the specific surface area and a surface-erosion mechanism was noted on enzymatic degradation. Due to the kinetics of hydrogel degradation and tissue growth are closely related [46,47], further study should be conducted to investigate the role of scaffold degradation and remodeling rates in assessing the performance of 3D-printed hydrogel scaffolds.

On account of the unique biological characteristics of stem cells, many reports have confirmed the great application prospect of stem cells in tissue engineering [32,48]. After successful fabrication of optimized scaffolds, the functional modification of marrow stem cells (MSC) homing ability was conducted for the regeneration ability and forming complex tissues [49,50,51]. However, the main reason hindering the effective translation of MSC homing therapy was that specific cells cannot be effectively selected to defect tissue [49]. In the present study, it was noted that the conjugating procedure had no influence on the bioactivity of BMSCs, and no cytotoxic compounds were produced, as evidenced by the cell viability and morphological alteration (Figure 3). Additionally, the fluorescent images of cells showed that BMSCs could maintain similar shapes in each specific PEGDA-GelMA-CSMA scaffolds (Figure 3). The images suggest chondrocyte-like round morphology after incubation in a chondrogenic induced culture medium (Figure 5 and Figure 6). The mean fluorescence intensity of F-actin indicated that the expression of F-actin was significantly upregulated with the increased amounts of CSMA, because CSMA could provide a biomimetic environment like the ECM of cartilage. Therefore, our findings demonstrated that the CSMA matrix in the PEGDA-GelMA-CSMA scaffolds could provide an environment for cell adhesion, proliferation, and differentiation.

In 3D-printed PEGDA-GelMA-CSMA scaffolds, the main chondrogenic marker of COL2 was upregulated in the RT-PCR and immunofluorescence tests (Figure 4 and Figure 5), whereas the main osteogenic marker genes, COL1 and ALP were downregulated compared to the PEGDA-GelMA scaffolds (Figure 4). The BMSCs on the PEGDA-GelMA scaffolds took on an elongated shape like fibroblasts, while the round chondrocyte-like cells on the PEGDA-GelMA-CSMA scaffold led to the decreased expression of osteogenesis markers of COL1 and ALP. This finding was consistent with a previous study, which illustrated that cell morphology was associated with cell differentiation [1]. The in vitro result was particularly relevant to clinical practices [51]. The incorporation of CSMA to the scaffolds resulted in increased accumulation of cartilage matrix molecules and increased compressive modulus. The expression of cartilage-specific genes (COL2, ACAN, SOX9, and PRG4) (Figure 4) and GAG/DNA ratio were higher in constructs containing CSMA (Figure 7), indicating that CSMA promoted biosynthesis of cartilage-specific matrix molecules.

Mechanical strength is an important metric to determine the quality of tissue cartilage. Studies have shown that the cartilage from bovine femoral condyles has Young’s modulus of approximately 0.3 MPa [52], and GAGs have a significant effect on the compression stiffness of the tissue through water absorption [53]. In this study, the high GAG level fully accounted for changes in the mechanical properties. The reason for the discrepancy in GAG content and construct modulus may be the favorable effect of PEGDA-GelMA-CSMA scaffolds: i.e., enhancing the matrix distribution. Additionally, the collagen network also facilitated improvement in the stiffness of the constructs. The significantly increased level of COL2 was induced by the CSMA hydrogel, indicating that CSMA enhanced the distribution of COL2 and ACAN. As a result, the mechanical properties were improved remarkably. The newly synthesized constructs containing CSMA should be more interconnected to exhibit greater mechanical reinforcement. The mechanisms by which CSMA enhances the distribution of new matrix and improves its mechanical properties are still not fully clear and warrant further investigation.

## 5. Conclusions

In summary, we prepared a novel class of 3D-printed PEGDA-GelMA-CSMA scaffolds for cartilage tissue regeneration. The findings showed that the higher content of CSMA possessed higher compressive modulus and slower degradation. The cell viability indicated these hydrogel scaffolds were non-toxic with good cytocompatibility. Cytoskeleton staining, immunofluorescence staining, and RT-PCR test verified that the PEGDA-GelMA-CSMA scaffolds could decrease the expression of osteogenic marker genes and enhance the expression of cartilage-specific genes as well as matrix deposition of the increased level of GAG. These customizable 3D-printed PEGDA-GelMA-CSMA scaffolds pave a new pathway for acquiring intelligent biomaterials for applications in cartilage tissue engineering.

## Figures and Tables

**Table 1:** Primer sequences are used in this study.

Gene	Forward Primers (5′-3′)	Reverse Primers (5′-3′)
*COL1*	ACCCCAGAAACAGACGACAAACAAC	ATGAATGCAACGGCAAAAACAAATC
*COL2*	GCAGCTGTGTGCAGGAGGGGAAG	TGGCAGTGGCGAGGTCAGTAGGG
*ACAN*	GACTCATTGTTAGAGGACAGCCA	CACTCCCAAAAAGAACTCCAGAT
*PRG4*	GGCAGGGAATGTGACTGTGATG	TGGGTGAGCGTTTAGTTGTTGA
*SOX9*	CGGCGGAGGAAGTCGGTGAAGA	AGTGGTGGGTGGGGTGGTGGTG
*ALP*	CCGCAAGTATATGTATCCCAAA	CCCAAGAGGTAGTCCACAGTGT
*GAPDH*	CATCAAGAAGGTGGTGAAGCAGG	AGCATCGAAGGTAGAGGAGTGGG

COL1: type I collagen; COL2: type II collagen; ACAN: aggrecan; PRG4: proteoglycan 4 precursors; SOX9: SRY-related high mobility group-box gene 9; ALP: alkaline phosphatase; GAPDH: glyceraldehyde-3-phosphate dehydrogenase.

**Table 2:** Composition and notation of 3D scaffolds tested in this study.

Composition (wt. %)	Notation
10% PEGDA, 15% GelMA	PEGDA10-GelMA15
10% PEGDA, 14% GelMA, 1% CSMA	PEGDA10-GelMA14-CSMA1
10% PEGDA, 12.5% GelMA, 2.5% CSMA	PEGDA10-GelMA12.5-CSMA2.5
10% PEGDA, 10% GelMA, 5% CSMA	PEGDA10-GelMA10-CSMA5

## Data Availability

All datasets generated for this study are included in the article.
